# Regulatory Efficacy of the Polyunsaturated Fatty Acids from Microalgae *Spirulina platensis* on Lipid Metabolism and Gut Microbiota in High-Fat Diet Rats

**DOI:** 10.3390/ijms19103075

**Published:** 2018-10-09

**Authors:** Tian-Tian Li, Yuan-Yuan Liu, Xu-Zhi Wan, Zi-Rui Huang, Bin Liu, Chao Zhao

**Affiliations:** 1 College of Food Science, Fujian Agriculture and Forestry University, Fuzhou 350002, China; litiantian_sweet@163.com (T.-T.L.); liuyyai@163.com (Y.-Y.L.); wxz951317@163.com (X.-Z.W.); inaccelworld@gmail.com (Z.-R.H.); 2 Fujian Province Key Laboratory for the Development of Bioactive Material from Marine Algae, Quanzhou Normal University, Quanzhou 362000, China; 3 Department of Chemistry, University of California, Davis, CA 95616, USA

**Keywords:** *Spirulina platensis*, polyunsaturated fatty acids, lipid metabolism, AMPK signal pathway, gut microbiota

## Abstract

Ultra-high performance liquid chromatography coupled with photo-diode array detector and electrospray ionization-mass spectrometry was employed to analyze the major fatty acids in *Spirulina platensis* 95% ethanol extract (SPL95). The effects of SPL95 on hepatoprotection were evaluated, including liver tissue histopathology, liver, and serum biochemical analysis. The active principle of SPL95 revealed a hypolipidemic effect, as indicated by down-regulating the mRNA and protein levels of sterol regulatory element-binding transcription factor-1c, 3-hydroxy-3-methyl glutaryl coenzyme A reductase, acetyl CoA carboxylase pathway, and upregulating adenosine 5′-monophosphate-activated protein kinase-α in liver. SPL95 enriched the beneficial bacteria, including *Prevotella*, *Alloprevotella*, *Porphyromonadaceae*, *Barnesiella*, and *Paraprevotella*. Treatment with SPL95 led to a decrease in microbes, such as *Turicibacter*, *Romboutsia*, *Phascolarctobacterium*, *Olsenella*, and *Clostridium XVIII*, which were positively correlated with serum triglyceride, total cholesterol, and low-density-lipoprotein cholesterol levels, but negatively correlated with the serum high-density-lipoprotein cholesterol levels. These results provide evidence that the fatty acid from SPL95 may be used as a novel adjuvant therapy and functional food to regulate gut microbiota in obese and diabetic individuals.

## 1. Introduction

Lipid metabolism disorder (LMD) is a risk factor for obesity, hyperlipidemia, hyperglycemia, fatty liver, cardiopathy, clinical syndrome, and another metabolic syndrome. The illnesses where trouble occurs in breaking down or synthesizing fats are characterized by high levels of triglyceride (TG), total cholesterol (TC), and low-density-lipoprotein cholesterol (LDL-c), coupled with low levels of high-density-lipoprotein cholesterol (HDL-c) [1]. The popularity of LMD has become a public concern, as most research on LMD tends to study cities and rich communities, while few studies involve nomadic low-income poor people. The International Diabetes Federation estimates that a quarter of adults globally have LMD [2]. In addition, the prevalence of Asian LMD is rising to a level similar to that of Western countries. Allopathic hypolipidemic drugs are available at large in the market, but with their own adverse effects and contraindications [3]. Despite significant advances in the development of frequently used lipid-lowering drugs, the side effects of these drugs are proportional to time. For example, statins are relatively common drugs that are toxic to multiple human organs. To a certain extent, these drugs can also cause liver failure, gastrointestinal dysfunction, and weakened body resistance. Recent studies have shown that dietary factors play a role in regulating metabolism, and making it a safe and economic way to treat LMD [4]. Therefore, the search for novel and natural cholesterol-lowering food components was extremely urgent. Algae have developed more and more functional foods [5,6]. *Spirulina platensis*, a microalga, has a special active ingredient formula [7]. It contains a lot of antioxidants, such as β-carotene, tocopherols, phycocyanin, microelements, and polyunsaturated fatty acids, especially γ-linolenic acid, and phenolic compounds. *Spirulina* is believed to be one of the most important and excellent healing and preventative nutrients in the 21st century for its comprehensive nutritional composition and remarkable therapeutic effect [8]. Therefore, among people with inflammatory diseases, insulin resistance, diabetes, and nonalcoholic fatty liver, *Spirulina* is recommended as a dietary supplement, and also has the effect of reducing drug toxicity [9].

The serine kinase adenosine 5’-monophosphate-activated protein kinase is an intracellular energy sensor that is a key factor that plays a role in regulating glucose and lipid metabolism [10]. Sterol regulatory element-binding transcription factor-1c (SREBP-1c) can preferentially enhance the transcription of genes required for fatty acid synthesis [11]. The 3-hydroxy-3-methyl glutaryl coenzyme A reductase (HMG-CoA), as the rate-limiting enzyme in cholesterol biosynthesis, is regulated through a negative feedback mechanism [12]. Acetyl CoA carboxylase (ACC), as the rate-limiting enzyme in de-novo lipogenesis, exists around the mitochondria and controls β-oxidation of fatty acids in the mitochondria [13].

Microbial species (*Firmicutes* and *Bacteroides*) associated with changes in blood lipids play a key role in the gut microbiota, which can also influence host energy and lipid metabolism by regulating serum lipid levels. It is widely believed that drugs can affect the state of the body by altering the microbiota of the gastrointestinal system. Moreover, ethanol extracts of many algae can change the gut microbiota [6,14]. In the present work, the hypolipidemic activity of the fatty acid in 95% ethanol extracts from *S. platensis* (SPL95) using in vivo high-fat diet rats was investigated. Its function of melioration of lipid metabolism through exploring body weight, serum index, and gut microbiota was determined. Molecular mechanisms of hypolipidemic effects of SPL95 were also identified.

## 2. Results

### 2.1. Characterization of Potent Major Compounds

The analysis of SPL95 resulted in the isolation of ten major components, eight of which were polyunsaturated fatty acids (Figure S1). The different chromatographic peaks appeared gradually during the retention time of 0.65 to 12.21 min, and then attempts were made to identify these components explicitly based on QTOF/MS (Figure S2; Table 1). MS/MS analysis has demonstrated the proposed presence of polyunsaturated fatty acids compounds. The specific structure of each peak was determined by comparing those MS spectral data reported in previous studies [15,16,17,18].

### 2.2. Effect of SPL95 on Body Weight and Serum Lipids of High-Fat-Diet Rats

**Figure 1:**
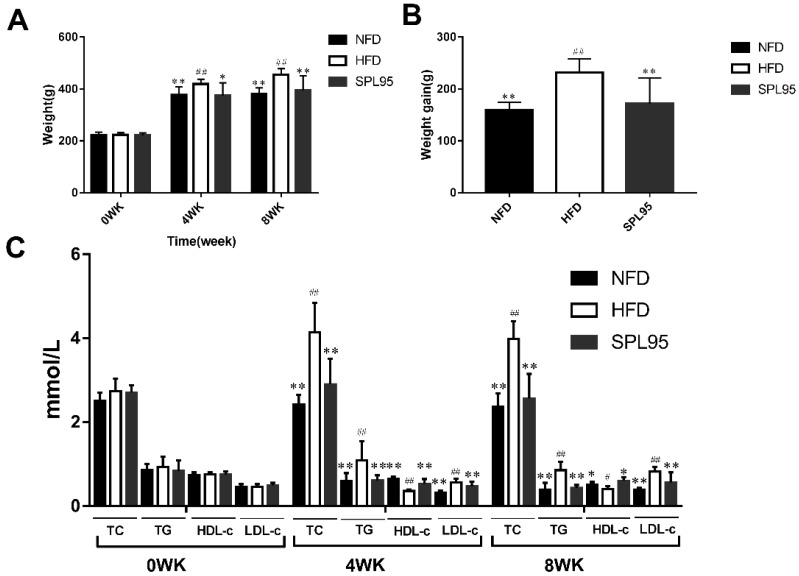
Effect of SPL95 on body weights (**A**), body weight gain (**B**), and serum lipid parameters (**C**) of high-fat-diet rats during the experimental period. NFD: Normal fat diet; HFD: High-fat diet; SPL95: 150 mg/(kg·day) *S. platensis* 95% ethanol extract; WK: Week. NFD group, rats fed NFD and gavaged with 150 mg/(kg·day) normal saline. HFD group, rats fed HFD and gavaged with 150 mg/(kg·day) normal saline. SPL95 group, rats fed HFD and gavaged with 150 mg/(kg·day) SPL95. Data are expressed as mean ± SD (*n* = 8). ^#^ *p* < 0.05 and ^##^ *p* < 0.01, compared with NFD group; * *p* <0.05 and ** *p* < 0.01, compared with HFD group. TG, triglyceride; TC, total cholesterol; HDL-c, high-density-lipoprotein cholesterol; LDL-c, low-density-lipoprotein cholesterol.

After a four-week experimental period, there was a statistically significant difference between the SPL95 group and high-fat-diet (HFD) group (*p* < 0.05) (Figure 1). After 8 weeks, all experimental rats gained weight. In particular, the average weight of rats in the HFD group was observably higher than that in the normal-fat-diet (NFD) group (*p* < 0.01). Compared with the high fat diet-induced HFD model, the SPL95 significantly reduced the body weight of rats after 8 weeks of treatment.

Moreover, the contents of serum TG, TC, HDL-c, and LDL-c in each experimental group were recorded. TG and TC concentrations of the SPL95-treated group were lowered significantly when compared to those of HFD (*p* < 0.01). SPL95 had decreased serum TG and TC levels by 49.41% and 35.68% compared to those of the HFD group after 8 weeks’ supplement, respectively. Similarly, the LDL-c levels in the SPL95 group were significantly lower than those of the HFD group after 4 and 8 weeks of treatment (*p* < 0.01). The HDL-c levels of the SPL95 treatment group were significantly increased by 47.22% (*p* < 0.01) and 50.00% (*p* < 0.05) compared to those of the HFD group at the end of the 4th or 8th week, respectively. The results showed that SPL95 could significantly meliorate serum lipid profiles (Figure 1C).

### 2.3. SPL95 Attenuates HFD-Induced Hepatic Steatosis

**Figure 2:**
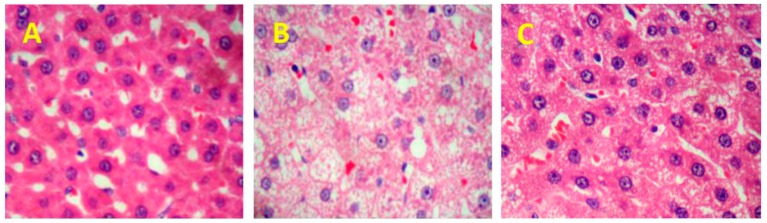
Histopathological analysis of rat hepatic tissues in the different groups at 40× magnification. (**A**) NFD group; (**B**) HFD group; (**C**) SPL95 group.

The liver histopathological examinations using hematoxylin and eosin (H&E) staining are shown in Figure 2. The hepatocyte nuclei were large and round in normal rats, which indicated the well functions (Figure 2A). By contrast, severe changes were exposed in the livers of HFD rats, such as ocal necrosis, congestion, and dilation of the central vein (Figure 2B). However, treatment of liver with SPL95 alleviated hepatocyte injury and inflammation in hyperlipemia rats except for a few fat droplets (Figure 2C), which showed that SPL95 has displayed a protective effect on the liver.

### 2.4. Effect of SPL95 on Liver Function

The liver lipid distribution was indicated in Table 2. TC, TG, LDL-c, and free fatty acids (FFA) levels were significantly increased in the HFD group over those in the NFD group. Meanwhile, HDL-c levels were decreased, which showed that the fatty liver rat model has been successfully established. The therapeutic effect of the SPL95-supplemented group was determined by a decrease in lipid parameters, such as reductions in TC, TG, LDL-c, alanine transaminase (ALT), aspartate transaminase (AST), and FFA levels (*p* < 0.01). Additionally, the ALT and AST levels in the liver were measured to reveal the effect of SPL95 supplementation on liver function in rats. As shown in Table 2, the concentrations of AST and ALT in the HFD group were significantly increased, indicating the rat model was a state of liver dysfunction. However, the SPL95 group significantly reduced these two parameters (*p* < 0.01), which showed that SPL95 can effectively improve liver function.

### 2.5. Effect of SPL95 on mRNA and Protein Levels Involved in Lipid and Glucose Metabolism

**Figure 3:**
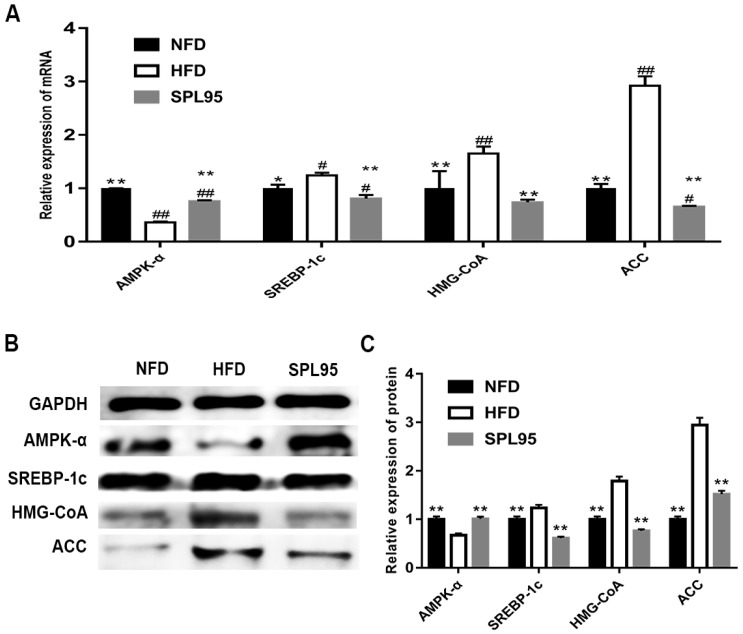
The mRNA and protein expressions levels involved in lipid and glucose metabolism as determined using real-time PCR (**A**) and western blotting (**B**,**C**). The differences were assessed by ANOVA and denoted as follows: ^#^ *p* < 0.05 and ^##^ *p* < 0.01 compared with the NFD group; * *p* < 0.05 and ** *p* < 0.01 compared with the HFD group.

Effects of SPL95 on adipogenic and glucose metabolism genes, including AMPK-α, SREBP-1c, HMG-CoA, and ACC, were investigated using RT-qPCR (Figure 3A). Additionally, western blot analysis was also used to assess protein expressions (Figure 3B,C). The transcription level of AMPK-α was significantly upregulated by SPL95 compared to that of HFD rats (*p* < 0.01), which was similar to that observed in NFD rats. SPL95 treatment could downregulate the mRNA expression of HMG-CoA, ACC, and SREBP-1c genes (*p* < 0.01). The SREBP-1, HMGCR, ACC (*p* < 0.01), and AMPK-α (*p* < 0.05) had the same trends at protein levels.

### 2.6. SPL95 Modulated Gut Microbiota of High-Fat-Diet Rats

**Figure 4:**
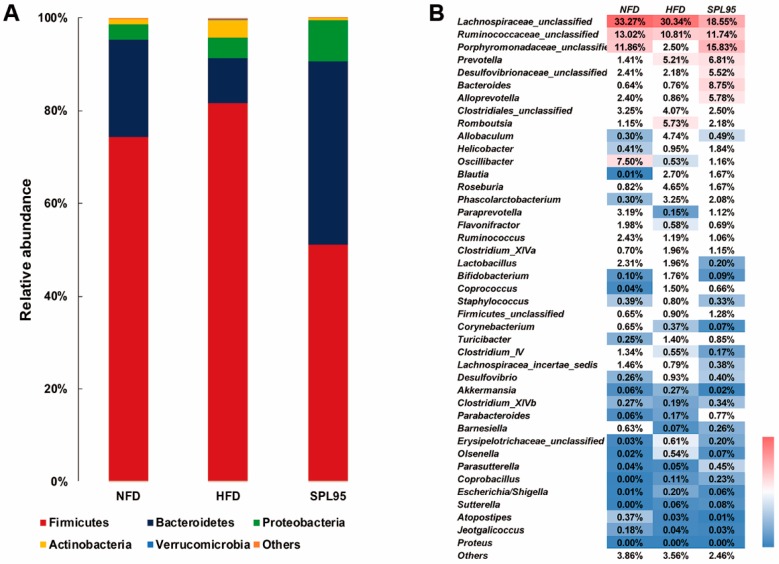
Changes in the bacterial composition of rat intestinal contents according to different genera, five rats were randomly selected from each experimental group for analysis of caecal microbiota. (**A**) Composition of gut microbiota at the phylum level. (**B**) Composition of gut microbiota at the genus level.

Comparing the relative abundances of bacterial taxa between groups after 8 weeks of treatment, Firmicutes, Bacteroidetes, Proteobacteria, Actinobacteria, and Verrucomicrobia were the major bacterial taxa detected in the rats’ intestinal contents at the level of phylum in all groups, whereas a small percentage belonged to unassigned phylum (Figure 4A). This had no significant effect on the relative abundance of different bacterial phyla at this period. However, Actinobacteria differed significantly in the supplementation of HFD. And HFD also induced a significant phylum-wide shift between Bacteroidetes (9.76%) and Firmicutes (81.51%), which was modified by SPL95 treatment during the active period. Firmicutes (57.09%) was significantly decreased and Bacteroidetes (33.75%) was significantly increased over time with SPL95 treatment, whereas the Verrucomicrobia population remained unaffected by NFD and SPL95. Finally, the abundant numbers of Verrucomicrobia also significantly increased with HFD.

At the genus levels, the presence of distinct bacterial taxa was also detected (Figure 4B). Through 8 weeks of treatment, great changes were made to the relative abundance of specific taxa, such as *Ruminococcaceae*, *Porphyromonadaceae*, *Prevotella*, *Desulfovibrionaceae*, *Bacteroides*, *Alloprevotella*, *Helicobacter*, *Paraprevotella*, *Oscillibacter*, and *Barnesiella*, which happened the most prominent increase after SPL95 administration at the genus level. Furthermore, the numbers of *Lachnospiraceae_unclassified*, *Romboutsia*, *Allobaculum*, *Roseburia*, *Clostridium_XlVa*, *Erysipelotrichaceae*, *Coprococcus*, *Turicibacter*, *Escherichia/Shigella*, *Clostridiales*, *Phascolarctobacterium*, and *Desulfovibrio* decreased in the intestinal contents of SPL95-fed rats compared to those of the HFD group.

### 2.7. Correlations of Biochemical Data and Key Phylotypes of Caecal Microbiota

**Figure 5:**
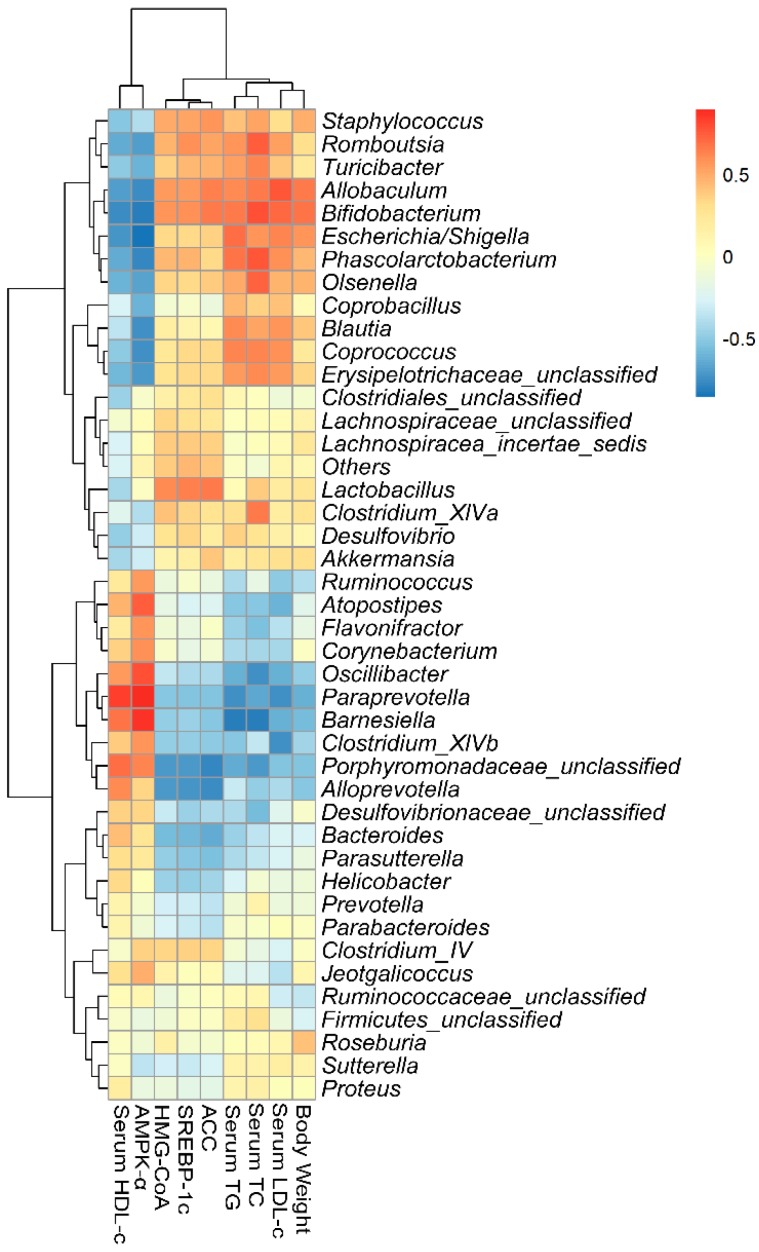
Statistical Spearman’s correlations between the caecal microbiota of significant differences and lipid metabolic parameters in SPL95, HFD, and NFD groups. The intensity of the color represents the degree of association between caecal microbiota of significant differences and MetS-associated parameters.

Spearman’s correlation analysis was performed to examine for a possible connection between the composition of intestinal microflora and lipid parameters in model rats at the genus level. *Turicibacter*, *Romboutsia*, *Phascolarctobacterium, Erysipelotrichaceae*, *Clostridium_XlVa*, and *Desulfovibrio* were significantly enriched in the HFD group. These strains were positively correlated with serum TG, TC, and LDL-c levels, or liver SREBP-1c, HMG-CoA, and ACC levels, while negatively with serum HDL-c and liver AMPK-α levels (Figure 5).

**Figure 6:**
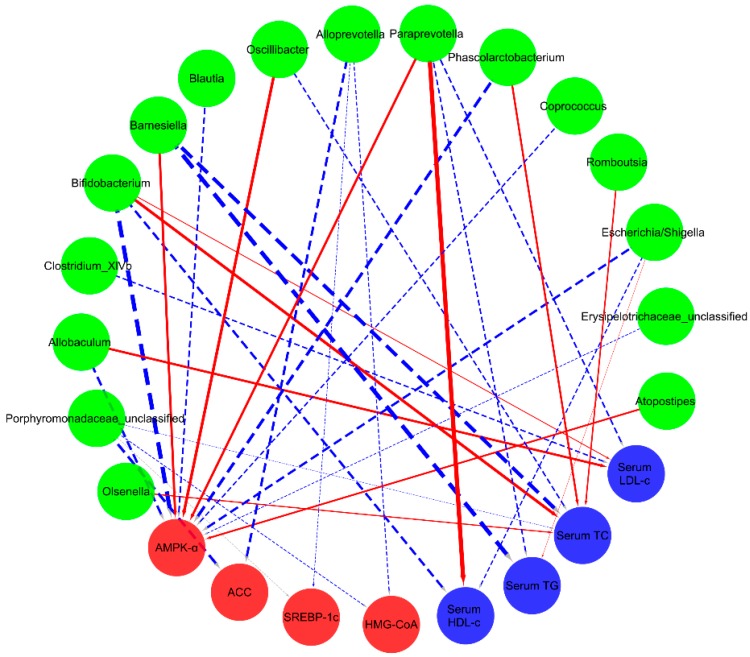
Visualization of the correlation network according to the partial correlation between the caecal microbiota of significant differences and the parameters associated with lipid metabolism disorder. Each node represents the gut microbiota genera and parameters associated with lipid metabolism disorder. The solid red line and dotted blue line represent positive and negative correlation, respectively. In addition, the line width indicates the strength of correlation. Only the significant edges are drawn in the network using the Spearman correlation test (|r| > 0.7, FDR adjusted *p* < 0.01).

More interestingly, the relative abundance of *Paraprevotella* showed positive relationships with serum HDL-c and liver AMPK-α. *Bifidobacterium* and *Escherichia/Shigella* were negatively correlated to serum HDL-c and liver AMPK-α (Figure 6). Meanwhile, the serum LDL-c index was found to have a positive relationship with the bacteria of *Allobaculum* and *Bifidobacterium*, and then had a significant negative correlation with *Paraprevotella*. Interestingly, on one hand, serum TC was positively correlated with *Olsenella*, *Bifidobacterium,* and *Phascolarctobacterium* to some extent; on the other hand, it was negatively correlated with *Barnesiella*, *Porphyromonadaceae*, *Oscillibacter*, and *Romboutsia*. Moreover, the relative abundances of *Porphyromonadaceae* and *Alloprevotella* revealed negative relationships with liver HMG-CoA, SREBP-1c, and ACC, which suggested that these bacteria were important factors in the beneficial effects of SPL95 on lipid metabolism regulation.

## 3. Discussion

Hyperlipidemia, hypercholesterolemia, intestinal microflora disorders, and intestinal barrier dysfunction are all associated with excessive intake of high fat and high glucose in the host. A long-term high-fat diet may lead to a significant increase in body weight, which is largely due to excessive food intake. However, the weight loss effect of SPL95 treatment was found to be significant in high-fat fed rats, which may be associated with enhanced energy metabolism. These presented data provided unique and novel insights into anti-hyperlipidemia and the lipid-lowering effects of the polyunsaturated fatty acids from from *S. platensis*. An abnormal increase in serum lipid content induces life-threatening diseases. Hyperlipidemia is one of the most common LMDs, characterized by high levels of TC, TG, and LDL-c, and low levels of HDL-c. Long-term intake of HFD is associated with metabolic syndrome in humans and rodents [19]. Interestingly, SPL95 treatment during the 8-week experimental period could stabilize high serum HDL-c levels and decreased TC, TG, and LDL-c levels. Epidemiological studies have shown that LDL-c and TG are thought to be associated with cardiovascular disease and play major roles in the development of treatment strategies. Decreased TG levels are generally positively associated with effective improvement of vascular disease [20]. The data showed that the SPL95 group can improve liver FFA, AST, and ALT levels in HFD rats. The hepatic steatosis markers TC, TG, and other hepatic indexes of SPL95 decreased, indicating the SPL95 could restore the high-fat diet induced fatty liver. Also, SPL95 would be used as a functional supplement for the treatment of hyperlipidemia. The biochemical alterations can be correlated with histological changes in the liver of rats. Feeding high-fat diets could lead to fat accumulation and massive accumulation of lipid droplets in the liver of rats. Hepatocytes steatosis and lipid droplets were obviously alleviated and decreased by SPL95 treatment, which indicated that *S. platensis* can reverse liver steatosis in rats.

SPL95 may act in lipid metabolism through activation of the AMPK signaling pathway. The AMPK can improve lipid metabolism disorders by regulating its own downstream factors [21], such as SREBP-1c, ACC, and HMG-CoA. With an activity in the promotion of lipogenesis, lipid homeostasis in cells is regulated by SREBPs. It also activates a number of genes specifically for the synthesis and uptake of TC, TG, and fatty acids in a special way. HMG-CR can increase the breakdown rate of plasma LDL. Atherosclerosis symptoms are induced by high LDL-c concentrations [22]. Internalization and degradation of LDL suppress HMG-CR in normal mammalian cells. HMG-CR causes the increase of plasma LDL-c levels, mainly through the production of cholesterol and other isoprenoids [23]. Phosphorylated AMPK inactivates ACC and lowers the intracellular malonyl-CoA level. Moreover, ACC produces malonyl CoA in the liver and controls β-oxidation of fatty acids in mitochondria, and it is also a substrate for fatty-acid synthesis and the rate-limiting enzyme of mitochondrial fatty-acid oxidation [24]. In this study, the expression of ACC in SPL95 was close to normal, probably due to the reduction of glucose and lipid levels. Another reason may be that ACC is inactivated after AMP activation, which inhibits fat production, a marker enzyme for lipid metabolism can be altered in HFD-induced obese rats by administration of SPL95. The decrease in serum parameters observed was partially caused by the downregulation of HMG-CoA expression by SPL95 supplementation. SREBP-1c, ACC, and HMG-CoA genes were found to significantly increase in the liver of HFD-fed rats with the decrease of AMPK-α which induced the accumulation of TC and TG. However, SPL95 treatment decreased the expression of SREBP-1c, ACC, and HMG-CoA. It has improved the AMPK-related signaling pathway in HFD-fed induced hyperlipidemic rats. Significant changes in gene expression associated with SREBP-1c may be responsible for lowering TG levels and inhibiting the synthesis of fatty acids [25]. The above results indicated that SPL95 ameliorated the fat synthesis-related genes in hyperlipemia rats by regulating the SREBP-1c and HMG-CoA reductase signaling pathway, providing compelling evidence for the potential application of SPL95 on hyperlipidemia.

**Figure 7:**
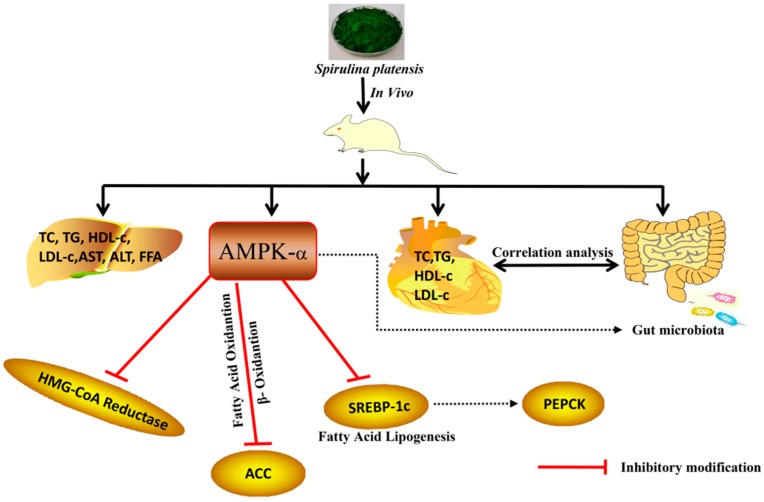
Summary of the mechanism of SPL95 to prevent LMD. Note: Stimulatory modification (green arrow), inhibitory modification (red arrow), and indirect modification (dotted arrow).

It is well known that the gastrointestinal microbial community plays key roles in human physiology and metabolism due to its multiple functions. These functions include extracting synthesizing vitamins, promoting intestinal homeostasis, improving blood cholesterol levels and effecting the development of atherosclerosis [26]. Intestinal microbiota can have a significant impact on blood cholesterol levels and the development of atherosclerosis. In experiment groups, rats were determined to explain the underlying mechanism for improving hyperlipidemia by *S.platensis* ethanol extract. Treatment with SPL95 increased the abundance of *Prevotella*, *Porphyromonadaceae*, *Barnesiella*, and *Paraprevotella*. *Prevotella* had a negative correlation with serum TG, TC, and LDL-c levels, but it had a positive correlation with serum HDL-c. Functional genomics indicated that P-type subjects had a higher resistance to LMD due to their higher carbohydrate digestion activity and lower bile acid biosynthesis activity [27]. The latest study has shown a positive correlation between bile acid and *Prevotella*, which was consistent with the results of this study. *Prevotella* regulates lipid levels by altering bile acid metabolism to change blood lipid levels. On the other hand, the *Barnesiella*, a family of *Porphyromonadaceae*, is part of the gut microbiota. In addition to the family *porphyrinaceae*, the *Bacteroidetes* include the family *Bacteroidaceae* and *Prevotellaceae*. Moreover, *Barnesiella* spp. regulates the composition of the microbiota and optimizes host survival [28]. Additionally, *Alloprevotella* and *Ruminococcus* were also enriched by SPL95 supplement. These bacteria are short chain fatty acid (SCFA) producers and negatively correlated with nonalcoholic fatty liver disease and LMD [29]. SCFA can be digested by the intestine and indirectly regulates energy metabolism and insulin sensitivity through specific receptors. Moreover, SPL95 has decreased the percentage of *Firmicutes* and increased the percentage of *Bacteroidetes* in cecal contents. These results were in accordance with the theory that body fat percentage correlates positively with the abundance of *Firmicutes* in the gut microbiota in humans and mice. In addition, changes in body weight and serum LDL-c were positively correlated with *Firmicutes*. There were increased proportions of *Porphyromonadaceae*, which was previously associated with nonalcoholic fatty liver disease, atherosclerosis, and diabetes [30]. In addition to increasing health-promoting bacteria, *Turicibacter* and *Clostridium XVIII* enriched in HFD were also decreased in the SPL95. Under this condition, alleviation of hyperlipidemia would be triggered. Moreover, *Clostridium XVIII* increased the gastrointestinal disorders and dysfunctions in hosts induced by obesity-related metabolic disorders or pro-inflammatory responses. The results revealed convincing data for the potential use of SPL95 in hyperlipidemia. Potent modulation of the intestinal microbiota during attenuation of metabolic disease was associated with its positive effects. The mechanism of SPL95 to lower blood lipid levels was briefly illustrated (Figure 7). Therefore, SPL95 may be beneficial for anti-hyperlipidemia and may reduce the risk of LMD.

## 4. Materials and Methods

### 4.1. Preparation of Spirulina platensis Extracts

The clean and air-dried *S. platensis* powder was obtained from King Dnarmsa *Spirulina* Co. Ltd. (Fuqing, China) and extracted using 95% ethanol at a ratio of 1:10 (*w*/*v*) at 45 °C for 0.5 h. The extract was then concentrated and freeze-dried. For gavage, the desired amount of the extract was weighed and for further study.

### 4.2. UPLC-QTOF-MS/MS Analysis of SPL95

Liquid chromatographic and mass spectrographic analysis were carried out on an ultra-performance liquid chromatography coupled with Waters quadrupole-time-of-flight tandem mass spectrometry (UPLC-Q-TOF-MS/MS) analyzer with C18 column (1.8 μm, 2.1 × 100 mm, USA) [31]. Solvent A (0.1% formic acid (*v*/*v*) in water) and solvent B (0.1% formic acid (*v*/*v*) in acetonitrile) were used as mobile phases according to the previous study [32]. The scan range was 50–1200 *m*/*z*, scan time 0.2 s, source offset 80, nebulizer gas flow (bar) 6.5, capillary voltage 2.0 kV at ESI^+^, ion source temperature 120 °C, desolvation temperature 450 °C, and nebulization gas flow 800 L/h at 800°C. The mass spectrometer and UPLC system were controlled by MassLynx 4.1 software (Waters, Millford, MA, USA).

### 4.3. Experimental Animals

Twenty-four healthy male Wistar rats were taken from the Shandong Laboratory Animal Center of Shandong Academy of Medical Sciences (Jinan, China) and kept in a temperature control room with 60 ± 5% relative humidity and given *ad libitum* to food and water. All animal experiments were in line with the Guiding Principles of the Animal Management and Use Committee of Fuzhou General Hospital (IACUC approval no. CGU11–119). After a week of acclimation period, rats were divided into the following three groups stochastically: Normal fat diet (NFD) group (rats fed NFD, *n* = 8), high-fat diet (HFD) group (rats fed HFD, *n* = 8), and SPL95 group (HFD-fed rats treated with SPL95, *n* = 8), using a method described previously [33]. Rats in the NFD group were given a basal diet (13.5% energy from fat; Lab Diet 5001; Lab Diet, Brentwood, MO, USA) and rats in the HFD and SPL95 groups were given a HFD (67% normal diet, 20% sugar, 10% lard, and 3% cholesterol). NFD and HFD groups were fed a basal diet and HFD with 2 mL 0.9% saline orally, respectively, while the SPL95 group was fed a HFD with 2 mL SPL95 extract (150 mg/kg·day) orally through gavage at the same time in the morning. According to the theory of traditional Chinese medicine, the crude drug dose of a 50 kg adult is 15 g, the rat’s crude drug dose is 5 times, and then the dose of 150 mg/kg can be obtained according to the actual extraction rate of *Spirulina* (10%).

### 4.4. Serum Samples Preparation

After 8 weeks of the experiment, rats were fasted for 12 h and anesthetized by intraperitoneal injection using ketamine hydrochloride. Blood was drawn through the heart and transferred to a centrifuge tube. Serum was separated at 5000 rpm for 10 min at 4 °C and then stored at −80 °C.

### 4.5. Liver Homogenate Preparation

The liver tissues were excised into several sections and then stored at −80 °C after snap-freezing. One hundred milligrams of liver tissue was mixed with 0.9 mL of saline and homogenized. After centrifugation at 8000 rpm for 15 min at 4 °C, the supernatant was taken for analysis.

### 4.6. Biochemical Assays

Determination of the level of TC, TG, HDL-c, LDL-c, alanine transaminase (ALT), aspartate transaminase (AST), and FFA in rats was done using assay kits (Nanjing Jiancheng Institute of Biotechnology, China) according to the instructions for the determination.

### 4.7. Liver Histopathological Analysis

The harvested liver samples were immediately fixed in 10% formalin solution and then treated with an ethanol solution. After paraffin embedding, the sections from the liver (5 μm) were used for pathological histology analysis through hematoxylin and eosin (H&E) staining at high magnification under an optical microscope (Nikon Eclipse TE2000-U, Nikon, Tokyo, Japan) [11].

### 4.8. Quantitative Reverse Transcription-PCR (RT-qPCR) Analysis

Total RNA was extracted from the liver tissues using an RNA extraction reagent and treated with a gDNA Eraser commercial kit (Takara, Kusatsu, Japan) to eliminate genomic DNA contamination. cDNA was synthesized using the PrimeScript^TM^ RT reagent Kit with a gDNA Eraser (Takara, Kusatsu, Japan). RT-qPCR of AMPK-α, SREBP-1c, HMG-CoA, ACC, and control β-actin was performed to monitor gene expression levels with the SYBR^®^ Premix Ex Taq™ II (Takara, Kusatsu, Japan). Following is a list of specific primers: AMPK-α, 5′-ATTTGCCCAGTTACCTCTTTCC-3′, R: 5′-GCTTGGTTCATTATTCTCCGAT-3′; SREBP-1c, F: 5′-GCTGTTGGCATCCTGCTATC-3′, R: 5′-TAGCTGGAAGTGACGGTGGT-3′); HMG-CR, F: 5′-AGTGGTGCGTCTTCCTCG-3′, R: 5′-CGAATCTGCTGGTGCTAT-3′); ACC, F: 5′-ACACTGGCTGGCTGGACAG-3′, R: 5′-CACACAACTCCCAACATGGTG-3′, and β-actin, F: 5′-ACGTCGACATCCGCAAAGACCTC-3′, R: 5′-TGATCTCCTTCTGCATCCGGTCA-3′. Amplifications were performed on an AB7300 Real-Time PCR system in the following thermal cycling condition: Programmed at 95 °C for 30 s and subjected to 40 cycles at 95 °C for 5 s, and 60 °C for 30 s [32,34].

### 4.9. Western Blot Analysis

Liver tissue was homogenized to obtain liver protein. The tissue proteins (20 μg) were separated on an SDS-PAGE gel and transferred onto PVDF membranes (Millipore, Billerica, MA, USA). The membranes were blocked overnight with no protein blocking solution (1% in 1 × TBS) and then incubated for 4 h using antibodies against HMGCR, SREBP-1c, AMPK-α, and GAPDH (Sangon, Shanghai, China). Immuno-reactive proteins were detected using the GeneGnome XRQ chemiluminescence Imaging System (SYNGENE, Cambridge, UK). The quantification of bands was determined by densitometric analysis using ImageJ densitometry software (National Institutes of Health, Bethesda, MD, USA).

### 4.10. Dynamic Profile of Intestinal Microflora in Response to SPL95

Caecal contents samples were collected at 8 weeks from different groups using a QIAamp-DNA stool mini kit (Qiagen, Hilden, Germany) to extract metagenomic DNA. The V3-V4 hypervariable domain of 16S rRNA gene was amplified using the universal primers (F: 5′-CCTACGGRRBGCASCAGKVRVGAAT-3′ and R: 5′-GGACTACNVGGGTWTCTAATCC-3′) [35]. Sequencing libraries were generated using the TruSeq^®^ DNA PCR-Free Sample Preparation Kit (Illumina, San Diego, CA, USA) following the manufacturer’s recommendations.

### 4.11. Bioinformatics Analysis

A high quality sequence was assigned to the sample based on the barcode. Denoise the effective sequence to study the diversity of species composition. Results were generated using Usearch (Version 7.1, http://drive5.com/uparse/) with 3% disagreement [36].

### 4.12. Statistical Analysis

Statistical analysis was performed using Data Processing System for Windows (version 2005.12.26; Hangzhou, China). Statistical significance was measured using one-way analysis of variance (ANOVA). Differences were deemed significant when *p* < 0.05. Relationships between gut microbiota composition and biochemical indicators in serum were determined using the Spearman’s rank correlation method.

## 5. Conclusions

Taken together, the polyunsaturated fatty acids from *S. platensis* were able to influence the lipid metabolism of Wistar rats fed with a high-fat diet. Histopathological analyses of the rat livers displayed that fatty acids from SPL95 reduced the incidence of liver lesions and improved hepatocyte abnormality. Moreover, its intervention provided a hypolipidemic effect by upregulating AMPK-α and downregulating SREBP-1c and HMG-CoA pathways in the liver. It regulated the gut microbiota structure by increasing the abundance of specific beneficial bacteria, including *Prevotella* and SCFA producers. Whereas the identification of ethanol extracts of *S. platensis* has rarely been investigated until now, the present study was of novel significance. Moreover, it characterized a new potential therapeutic role of SPL95 and conducted an in-depth analysis of changes in intestinal flora and molecular mechanisms upon SPL95 supplementation for the first time.

## Figures and Tables

**Table 1:** Characterization of probable major metabolites of ethanol extract from *Spirulina platensis* by UPLC/Q–TOF–MS.

No.	Rt (min)	Compound Name	Probable Formula	Measured [M + H]+ (*m*/*z*)	Representative Fragmentation	References
1	0.65	Octadecatetraenoic acid	C_18_H_28_O_2_	277	205.9875, 277.090, 278.0923	[15,16,17,18]
2	1.88	Heptadecane	C_17_H_36_	239	220.0831, 238.0942, 239.0961, 739.2668	
3	2.82	Methyl stearate	C_19_H_38_O_2_	298	136.0620, 137.0634, 298.0974	
4	3.37	Unknown	C_14_H_2_O_2_	220	105.0445, 202.0720, 220.0829, 221.0849	
5	4.66	Gluconic acid	C_6_H_12_O_7_	197	133.1008, 179.1064, 197.1175, 251.0361	
6	7.51	Hexose + glycerol + palmitoleic acid	C_25_H_47_O_9_	491	467.2029, 468.2115, 490.1934, 491.1964	
7	9.92	Unknown		509	438.1961, 509.2724, 510.2752, 511.2782	
8	11.28	Hexose + Sulfoquinovosyl diacylglycerol + γ-linolenic acid	C_31_H_50_O_14_S	678	537.3036, 677.3709, 678.3750	
9	11.42	Hydroperoxy octadecatrienoic acid	C_18_H_30_O_4_	311	311.2577, 312.2608, 513.3030	
10	12.21	Sulfoquinovosyl diacylglycerol + linoleic acid	C_28_H_52_O_12_S	612	515.3187, 516.3215, 517.3237, 611.2857	

**Table 2:** Effect of SPL95 on TC, TG, HDL-c, LDL-c, ALT, AST, and FFA levels in the liver of rats.

Group	TC (mmol/L)	TG (mmol/L)	HDL-c (mmol/L)	LDL-c (mmol/L)	ALT (mmol/L)	AST (mmol/L)	FFA (mmol/L)
NFD	1.61 ± 0.30 **	0.70 ± 0.05 **	0.91 ± 0.09 *	0.49 ± 0.04 *	26.89 ± 6.43 **	26.12 ± 4.06 **	264.51 ± 63.17 **
HFD	3.59 ± 0.72 ^##^	1.61 ± 0.08 ^##^	0.39 ± 0.23 ^#^	0.77 ± 0.16 ^#^	80.02 ± 10.32 ^##^	62.18 ± 10.28 ^##^	891.92 ± 222.59 ^##^
SPL95	1.63 ± 0.13 **	1.26 ± 0.33 **^##^	0.91 ± 0.25 **	0.42 ± 0.07 **	27.34 ± 3.14 **	29.57 ± 7.77 **	247.40 ± 68.94 **

Note: TG, triglyceride; TC, total cholesterol; HDL-c, high-density-lipoprotein cholesterol; LDL-c, low-density-lipoprotein cholesterol. ALT, alanine transaminase; AST, aspartate transaminase; FFA, free fatty acids. ^#^ *p* < 0.05 and ^##^ *p* < 0.01 compared with the NFD group; * *p* < 0.05 and ** *p* < 0.01 compared with the HFD group.

## Supplementary Materials

Supplementary materials can be found at http://www.mdpi.com/1422-0067/19/10/3075/s1.

## References

[1] Yuan F., Dong H., Fang K., Gong J., Lu F.E. (2017). Effects of green tea on lipid metabolism in overweight or obese people: A meta-analysis of randomized controlled trials. Mol. Nutr. Food Res..

[2] Sugimoto T., Sato M., Dehle F.C., Brnabic A.J.M., Weston A., Burge R. (2016). Lifestyle-related metabolic disorders, osteoporosis, and fracture risk in Asia: A systematic review. Value Health Reg. Issues.

[3] Waness A., Bahlas S., Al S.S. (2008). Simvastatin-induced rhabdomyolysis and acute renal injury. Blood Purif..

[4] Sarin S., Kaman L., Dahiya D., Behera A., Medhi B., Chawla Y. (2016). Effects of preoperative statin on liver reperfusion injury in major hepatic resection: A pilot study. Updates Surg..

[5] Zhao C., Wu Y.J., Yang C.F., Liu B., Huang Y.F. (2015). Hypotensive, hypoglycaemic and hypolipidaemic effects of bioactive compounds from microalgae and marine micro-organisms. Int. J. Food Sci. Technol..

[6] Zhao C., Yang C.F., Liu B., Lin L., Sarker S.D., Nahar L., Yu H., Cao H., Xiao J.B. (2018). Bioactive compounds from marine macroalgae and their hypoglycemic benefits. Trends Food Sci. Technol..

[7] Deng R.T., Chow T.J. (2010). Hypolipidemic, antioxidant, and antiinflammatory activities of microalgae *Spirulina*. Cardiovasc. Ther..

[8] Serban M.C., Sahebkar A., Dragan S., Stoichescu-Hogea G., Ursoniu S., Andrica F., Banach M. (2016). Systematic review and meta-analysis of the impact of *Spirulina* supplementation on plasma lipid concentrations. Clin. Nutr..

[9] Neyrinck A.M., Taminiau B., Walgrave H., Daube G., Cani P.D., Bindels L.B., Delzenneet N.M. (2017). *Spirulina* protects against hepatic inflammation in aging: An effect related to the modulation of the gut microbiota?. Nutrients..

[10] Grahame Hardie D. (2014). AMP-activated protein kinase: A key regulator of energy balance with many roles in human disease. J. Intern. Med..

[11] Cheng H., Xu N., Zhao W.X., Su J.J., Liang M.R., Xie Z.W., Wu X.L., Li Q.L. (2017). (-)-Epicatechin regulates blood lipids and attenuates hepatic steatosis in rats fed high-fat diet. Mol. Nutr. Food Res..

[12] Deboseboyd R.A. (2008). Feedback regulation of cholesterol synthesis: Sterol-accelerated ubiquitination and degradation of HMG-CoA reductase. Cell Res..

[13] Munday M.R. (2002). Regulation of mammalian acetyl-CoA carboxylase. Biochem. Soc. Trans..

[14] Zhao C., Yang C.F., Wai S.T.C., Zhang Y.B., Portillo M.Y., Paoli P., Wu Y.J., Cheang W.S., Liu B., Carpéné C. (2018). Regulation of glucose metabolism by bioactive phytochemicals for the management of type 2 diabetes mellitus. Crit. Rev. Food Sci..

[15] Feng H., Zhang B., He Z., Wang S., Salih O., Wang Q. (2018). Study on Co-Liquefaction of *Spirulina* and *Spartina* alterniflora in ethanol-water co-solvent for bio-oil. Energy.

[16] Pignitter M., Stolze K., Jirsa F., Gille L., Goodman B.A., Somoza V. (2015). Effect of copper on fatty acid profiles in non-and semifermented teas analyzed by LC-MS-based nontargeted screening. J. Agric. Food Chem..

[17] Herrero M., Vicente M.J., Cifuentes A., Ibanez E. (2010). Characterization by high-performance liquid chromatography/electrospray ionization quadrupole time-of-flight mass spectrometry of the lipid fraction of *Spirulina platensis* pressurized ethanol extract. Rapid Commun. Mass Spectrom..

[18] Karim A.A., Azlan A., Ismail A., Hashim P., Gani S.S.A., Zainudin B.H., Abdullah N.A. (2014). Phenolic composition, antioxidant, anti-wrinkles and tyrosinase inhibitory activities of cocoa pod extract. BMC Complement. Altern. Med..

[19] Buettner R., Parhofer K.G., Woenckhaus M., Wrede C.E., Kunz-Schughart L.A., Schölmerich J., Bollheimer L.C. (2006). Defining high-fat-diet rat models: Metabolic and molecular effects of different fat types. J. Mol. Endocrinol..

[20] Koyama Y., Maebara Y., Hayashi M., Nagae R., Tokuyama S., Michinaga S. (2012). Endothelins reciprocally regulate VEGF-A and angiopoietin-1 production in cultured rat astrocytes: Implications on astrocytic proliferation. Glia.

[21] Kim J.S., Ha T.Y., Kim S., Lee S.J., Ahn J. (2017). Red paprika (*Capsicum annuum L*.) and its main carotenoid capsanthin ameliorate impaired lipid metabolism in the liver and adipose tissue of high-fat diet-induced obese mice. J. Funct. Foods.

[22] Tobert J.A. (2003). Lovastatin and beyond: The history of the HMG-CoA reductase inhibitors. Nat. Rev. Drug Discov..

[23] Zou Z.Y., Hu Y.R., Ma H., Feng M., Li X.G., Ye X. (2015). Epiberberine reduces serum cholesterol in diet-induced dyslipidemia Syrian golden hamsters via network pathways involving cholesterol metabolism. Eur. J. Pharmacol..

[24] Brahma Naidu P., Uddandrao V.V.S., Ravindar Naik R., Suresh P., Meriga B., Begum M.S., Pandiyan R., Saravanan G. (2016). Ameliorative potential of gingerol: Promising modulation of inflammatory factors and lipid marker enzymes expressions in HFD induced obesity in rats. Mol. Cell. Endocrinol..

[25] Horton J.D., Goldstein J.L., Brown M.S. (2002). Srebps: Activators of the complete program of cholesterol and fatty acid synthesis in the liver. J. Clin. Investig..

[26] Dolan K.T., Chang E.B. (2016). Diet, gut microbes, and the pathogenesis of inflammatory bowel diseases. Mol. Nutr. Food Res..

[27] Nakayama J., Watanabe K., Jiang J., Matsuda K., Chao S.H., Haryono P., La-Ongkham O., Sarwoko M.A., Sujaya I.N., Zhao L. (2015). Diversity in gut bacterial community of school-age children in Asia. Sci. Rep..

[28] Ubeda C., Bucci V., Caballero S., Djukovic A., Toussaint N.C., Equinda M., Lipuma L., Ling L., Gobourne A., No D. (2013). Intestinal microbiota containing *Barnesiella* species cures vancomycin-resistant *Enterococcus faecium* colonization. Infect. Immun..

[29] Shang Q.S., Song G.R., Zhang M.F., Shi J.J., Xu C.Y., Hao J.J., Li G.Y., Yu G.L. (2017). Dietary fucoidan improves metabolic syndrome in association with increased *Akkermansia* population in the gut microbiota of high-fat diet-fed mice. J. Funct. Foods..

[30] Henao-Mejia J., Elinav E., Jin C., Hao L., Mehal W.Z., Strowig T., Thaiss C.A., Kau A.L., Eisenbarth S.C., Jurczak M.J. (2012). Inflammasome-mediated dysbiosis regulates progression of NAFLD and obesity. Nature.

[31] Zhang Y., Sun S.Q., Xing X.B., Du Z.X., Guo Q.Z., Yu W.L. (2016). The detection and identification of leachables in vaccine from plastic packaging materials using UPLC-QTOF MS with self-built polymer additives library. Anal. Chem..

[32] Huang Z.R., Zhou W.B., Yang X.L., Tong A.J., Hong J.L., Jia R.B., Lin J., Li T.T., Pan Y.Y., Lv X.C. (2018). The regulation mechanisms of soluble starch and glycerol for production of azaphilone pigments in *Monascus purpureus* FAFU618 as revealed by comparative proteomic and transcriptional analyses. Food Res. Int..

[33] Luo Y.C., Chen G., Li B., Ji B.P., Guo Y., Tian F. (2009). Evaluation of antioxidative and hypolipidemic properties of a novel functional diet formulation of *Auricularia auricula* and Hawthorn. Innov. Food Sci. Emerg. Technol..

[34] Zhao C., Yang C.F., Chen M.J., Lv X.C., Liu B., Yi L.Z., Cornara L., Wei M.C., Yang Y.C., Tundis R. (2018). Regulatory efficacy of brown seaweed *Lessonia nigrescens* extract on the gene expression profile and intestinal microflora in type 2 diabetic mice. Mol. Nutr. Food Res..

[35] Li K.K., Zhuo C., Teng C.Y., Yu S.M., Wang X., Hu Y., Ren G.M., Yu M., Qu J.J. (2016). Effects of *Ganoderma lucidum* polysaccharides on chronic pancreatitis and intestinal microbiota in mice. Int. J. Biol. Macromol..

[36] Henschel A., Anwar M.Z., Manohar V. (2015). Comprehensive meta-analysis of ontology annotated 16S rRNA profiles identifies beta diversity clusters of environmental bacterial communities. PLoS Comput. Biol..

