# Supplementary material for: Regulatory Efficacy of the Polyunsaturated Fatty Acids from Microalgae Spirulina platensis on Lipid Metabolism and Gut Microbiota in High-Fat Diet Rats
Source: Int J Mol Sci. 2018 Oct 9;19(10):3075. doi: 10.3390/ijms19103075 (PMC6213792; doi:10.3390/ijms19103075)
Supplement: Supplementary file 1 [file ijms-19-03075-s001.pdf]

Regulatory efficacy of the polyunsaturated fatty acids from microalgae *Spirulina platensis* on lipid metabolism and gut microbiota in high-fat diet rats

Tian-Tian Li[a#], Yuanyuan Liu[a#], Xu-Zhi Wan[a], Zi-Rui Huang[a], Bin Liu[a*], Chao Zhao[a, b, c*]

[a] College of Food Science, Fujian Agriculture and Forestry University, Fuzhou 350002, China
[b] Fujian Province Key Laboratory for the Development of Bioactive Material from Marine Algae, Quanzhou Normal University, Quanzhou 362000, China
[c] Department of Chemistry, University of California, Davis 95616, USA
[#]Tian-tian Li and Yuanyuan Liu contributed equally to this study.

*To whom correspondence should be addressed: No.15 Shangxiadian Rd., Fuzhou 350002, China; E-mail: binliu618@163.com (B. Liu), zhchao@live.cn (C. Zhao)

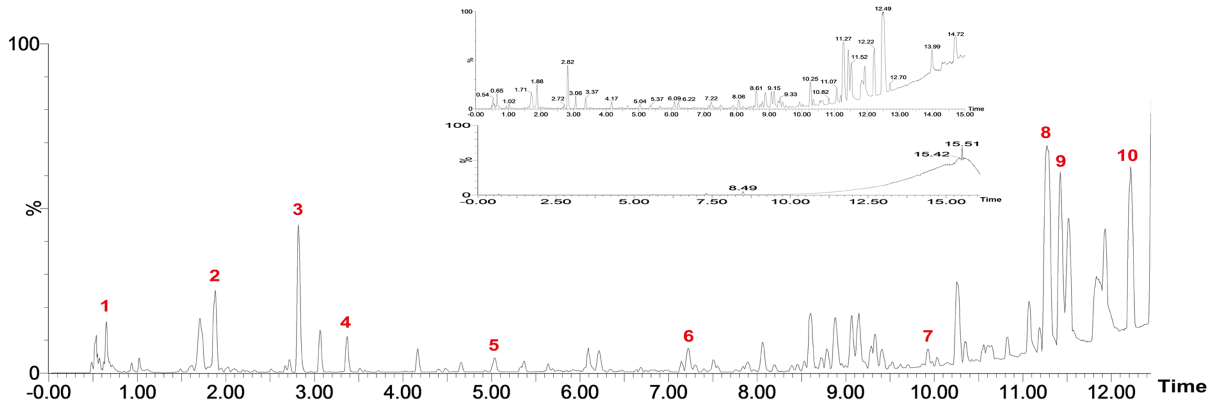

Supplementary Figure S1. Chromatographic peaks of *Spirulina platensis* ethanol extract in UPLC.

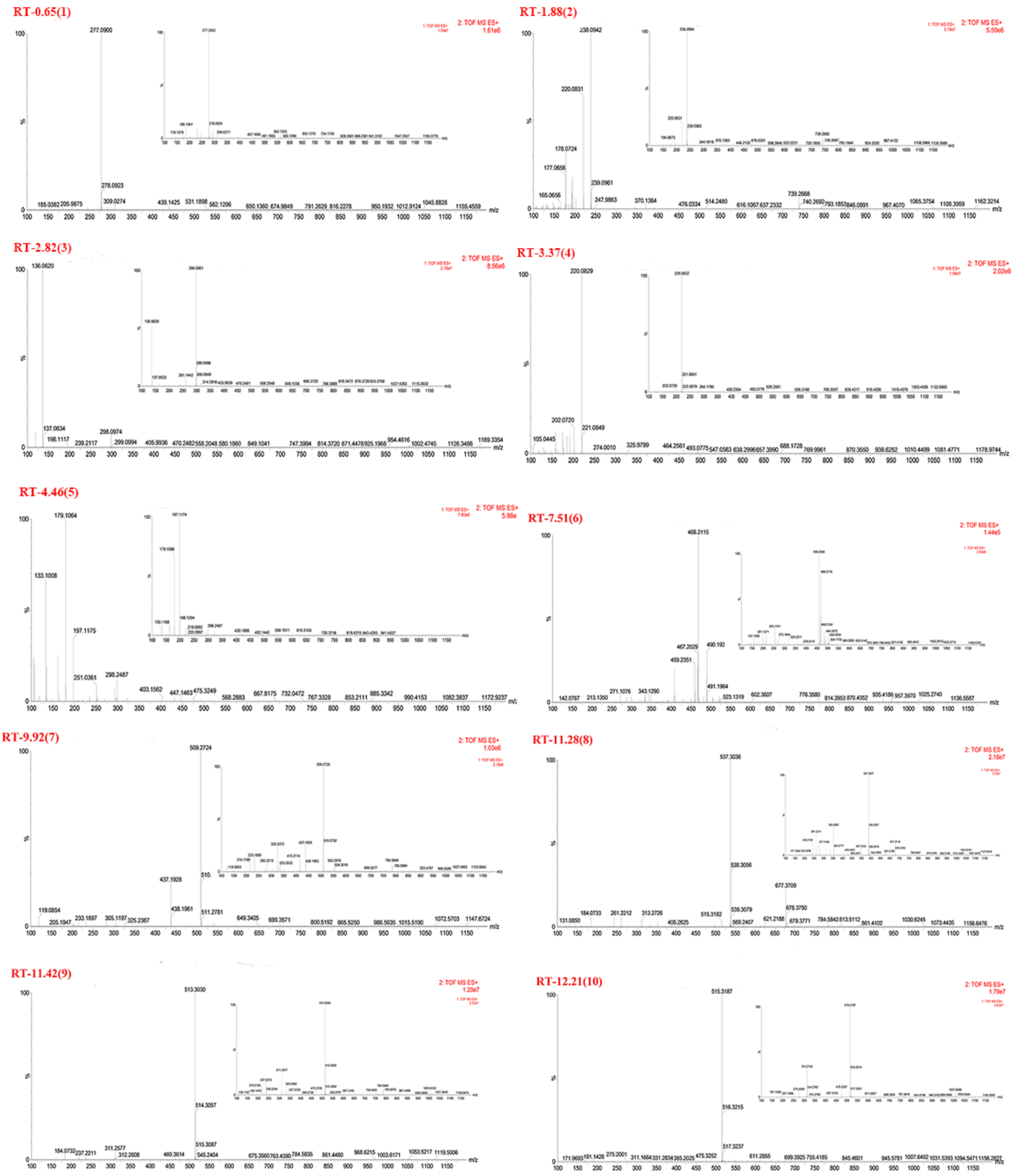

Supplementary Figure S2. Representative UPLC/Q-TOF MS chromatographs of ethanol extracts of *Spirulina platensis*.
